# Perspectives on the quality of climate information for adaptation decision support

**DOI:** 10.1007/s10584-024-03823-1

**Published:** 2024-11-05

**Authors:** Marina Baldissera Pacchetti, Suraje Dessai, James S. Risbey, David A. Stainforth, Erica Thompson

**Affiliations:** 1 Department of Science, Technology, Engineering and Public Policy, UCL, London, UK; 2 Earth Science Department, Barcelona Supercomputing Center, Barcelona, Spain; 3 School of Earth and Environment, University of Leeds, Leeds, UK; 4 Priestley Centre for Climate Futures, University of Leeds, Leeds, UK; 5 CSIRO, Hobart, Australia; 6 Grantham Institute for Climate Change and the Environment, LSE, London, UK; 7 Department of Physics, University of Warwick, Warwick, UK

**Keywords:** Climate information, Adaptation, Decision support, Projections, Storylines

## Abstract

We summarise the contributions to the Topical Collection on quality of climate information for adaptation decision support. Based on these contributions, we draw some further lessons for the development of high-quality climate information and services, bridging between a “credibility-first” paradigm (exemplified by top-down information provision from systematic downscaling or impact projections) and a “salience-first” paradigm (exemplified by user-led tailored information products or storylines) by looking to identify their respective strengths and use cases. We emphasise that a more nuanced collective understanding of the dimensions of information quality in climate information and services would be beneficial to users and providers and ultimately support more confident and effective climate adaptation decisions and policy-making.

## Introduction

The climate change commitment means that adaptation is unavoidable and increasingly urgent (Moss et al. 2013). Scientific knowledge about climate and climate change that is used in adaptation planning and decision-making should be of the highest attainable quality (e.g. Lu 2011; Wilby et al. 2009). However, past research has only addressed what “quality” means within disciplinary boundaries and by addressing methodological and conceptual issues in climate model assessments, mostly at a global scale (see Zeng et al. 2019; Krysanova et al. 2018; Parker and Risbey 2015; Lemos and Morehouse 2005, Cash et al. 2003).

The type of climate information and services needed for adaptation, however, is becoming increasingly diversified. It is largely regional and local in scale (e.g., Nissan et al. 2019), and it is derived using an increasingly diverse number of approaches. There are now a plethora of data, models and methods available to produce regional climate information. There are also an increasingly large number of climate service providers, both public and private, that cater to the growing information needs of sectors planning for a changing climate. Although research efforts have been made (Baldissera Pachetti et al. 2021b), there is as yet no widely accepted comprehensive framework to assess the quality of these data, models and methods. Such a framework is important to support trust in scientific output, as well as make sure that information is developed and interpreted in a way that mitigates the possibility of maladaptation.

The aim of the Topical Collection on “Perspectives on the quality of climate information for adaptation decision support” is to provide a multi- and inter-disciplinary selection of views of what counts as good scientific knowledge about future climate by drawing insights from philosophy of science, environmental social science and physical climate science. By “scientific knowledge about future climate” we mean any scientific statement or estimate (e.g. model- or data-derived statements, expert judgement, storylines, etc.) about the future state of the climate that is used to inform climate change adaptation, usually at the regional or local scale.

There are two main themes that guide the organisation of the contributions:

The **epistemic theme**, which explores questions related to the justification of scientific knowledge claims relevant for climate change adaptation. Relevant questions are, for example: What are adequate sources of scientific knowledge about future climate? How should these sources be evaluated, and how should we aggregate them?

The **practice-oriented theme**, which explores questions regarding the applicability of climate information for adaptation. Relevant questions are, for example: How are climate forecasts and projections delivered, and how are they received? How can we make climate information relevant, practical and applicable for adaptation?

By integrating interdisciplinary perspectives on these complementary themes relevant for the quality of climate knowledge for climate change adaptation, this Topical Collection provides a novel outlook on one of the most pressing challenges to climate change adaptation: the production of actionable scientific knowledge about future climate at regional and local scales.

This special issue is based on an interdisciplinary workshop that was held online in October 2020 as part of activities related to a project funded by the ESRC Centre For Climate Change Economics and Policy (https://www.cccep.ac.uk). The workshop was originally planned in person, and involved mostly UK-based researchers that the organizers understood were interested in the epistemic foundations of usable climate science. However, the workshop disrupted due to COVID, and a few international researchers were able to participate in the new online version. The workshop has shown that there is an urgent need for the development of an interdisciplinary discussion on the foundational issues of assessing quality for climate information that intends to inform adaptation decisions, which are still relevant at the time of writing of this editorial. The Topical Collection lays the foundations for future research on this topic, which is explored further in this editorial.

## Emerging good practices and challenges

The contributions to this Topical Collection provide an initial perspective on good practices and challenges, both foundational and practical, that arise when raising issues of quality of forward looking scientific information developed and used in climate change adaptation planning. There are three themes that emerge from the contributions that we discuss in a broader context and which raise issues and challenges for future research and action. The first two themes correspond to our original division into epistemic considerations, related to the concept of quality (Sect. 2.1), and practice-oriented considerations about how the quality of climate services can be improved (Sect. 2.2). We then identify a third theme: scientific quantification of uncertainties (Sect. 2.3).

### Epistemic considerations: What do we mean by quality of climate information?

The first paper in our topical collection (Baldissera Pacchetti et al. 2021a tests a framework for assessing the quality of regional climate information drawn from previous work by the same authors (Baldissera Pacchetti et al. 2021b) on the UK Climate Projections 2018 (UKCP18). The framework assesses climate projection quality along dimensions of transparency, theory, diversity, independence, comprehensiveness of evidence, and empirical adequacy. The application of the framework to highlights a lack of transparency and a lack of systematic approaches to characterising uncertainty in UKCP18. These issues are compounded for users of the climate information who are unaware of the sources of uncertainties associated with the projections. The analysis highlights the need to incorporate user perspectives more explicitly in the production of climate information, and to extend the set of approaches used to develop regional climate information of higher quality.

Taking a similar approach, Jebeile (2024) challenges the assumption that higher resolution climate information from regional climate models (RCMs) will result in more usable information. She applies the framework of credibility, salience, and legitimacy (Cash et al. 2003) - all of which are needed for usable information - to consider whether RCM information will indeed be usable, identifying a trade-off between credibility and salience. Credibility is largely epistemic and a function of the level of scientific confidence in an output. Salience is about whether information meets the needs of end users, and requires information to be local, complete, relevant and intelligible. Meeting the needs of end users in this way tends to entail the generation of additional uncertainties: Jebeile discusses the example of the nationally produced Swiss climate change scenarios (CH2018) and characterises a cascade of (increasing) uncertainty from general circulation models (GCMs) through regional simulation and downscaling approaches to impact models and finally communication choices. At each step the information becomes more salient - closer to what (some) users would need for decision support - but less credible due to the propagation of large uncertainties. Jebeile touches on some potential resolutions to this trade-off through approaches to decision-making under uncertainty, such as robust decision making.

Wilby and Lu (2022) take a perspective of the participants rather than the process, and develop a “tailoring” metaphor for climate services, distinguishing between three kinds of climate service customers who are respectively looking to buy a product which is ‘off-the-peg’, ‘outsourced’, or ‘bespoke’ according to the degree of interaction they require (or can afford to pay for). They posit that a better “shopping experience” is needed for potential customers, to ensure that the right kind of information is sought and provided. Their table of useful questions and discussion prompts will be of great value both to such potential customers and also to information providers seeking to ensure that their product is received and used appropriately.

These three contributions raise the question of how to understand and develop a concept of quality that is comprehensive and satisfactory as climate information is developed, translated and delivered for decision making in the context of climate change adaptation. Their concepts of quality are not identical, but are similarly rooted in the need for climate projections to be actually useful to the end users of any information product or climate service - and they each emphasise that scientific credibility alone is not sufficient for usefulness or useability.

### Practical considerations: How can we improve the quality of climate services?

There is an increasingly large number of climate service providers, ranging from public ones (including the national providers described above, and multinational institutions such as Copernicus Climate Change Service (C3S)) to private ones (e.g. Ramboll). Within this Topical Collection, both Baldissera Pachetti et al. and Jebeile have considered specific examples of national climate service provision. Baldissera Pachetti et al. identify key practical opportunities to improve UKCP18 through greater transparency - which in their definition has elements of both credibility and legitimacy - and through a more nuanced methodological approach related to embracing the subjective nature of model-derived probabilities, which we discuss further below. Reaching a similar conclusion, Jebeile identifies an opportunity to resolve trade-offs between salience and credibility in the Swiss climate change scenarios CH2018 by exploring decision-making approaches which are less sensitive to uncertainty.

Turning to the experience of providers themselves, Bojovic et al. (2022) present a discussion based on semi-structured interviews with seasonal forecast providers about the aims and challenges of these organisations. Within the framework of their defined role, these Global Producing Centres for Long-Range Forecasts (GPCs-LRF) are subject to common pressures of standardisation, open data, and significantly increasing user demands, but experience locally different expectations to provide or contribute to national, regional and global forecasting products. Taking the framework of credibility, salience, and legitimacy of Cash et al. (2003), the authors reflect on some challenges for GPCs-LRF and how they could improve the effectiveness of forecast production, provision, and communication. They highlight lack of forecast skill (within the dimension of credibility) as a particular challenge, requiring improvements in scientific capability, clearer definitions of quality that are relevant to users, and better communication. To address these challenges, the authors propose that an integrative and transdisciplinary approach will be needed to break down silos and ensure full participation of an ever-expanding stakeholder community.

Between the above contributions, we can start to see the outline of a coherent agenda for identifying and improving quality in climate services, an endeavour increasingly taken up by the climate services community (e.g., *Climateurope2*, *UK Climate Resilience Programme*). However, this community is still highly fragmented, and we still need to build bridges across different multidisciplinary subcommunities to create shared goals and values that are conducive to developing a standard for quality. In particular, there is a need to reconcile data- and prediction-oriented metrics of quality with more outcome- and user-oriented metrics. Data- and prediction-oriented metrics prioritise credibility and are usually easier to measure and provide. Outcome- and user-oriented metrics prioritise salience, and perhaps more closely demonstrate the actual value of climate service provision (Findlater et al. 2021).

### Scientific considerations: how should scientists quantify climate uncertainties?

Baldissera Pachetti et al. (2021a) and Jebeile (2024) have already identified that methodological questions about the quantification of uncertainty are not fully answered by UKCP18 and CH2018 climate projections. But it cannot be expected that climate service users will all have the technical capacity to discuss such methodological questions with scientific providers. As such, answering these questions within the scientific community will be necessary. Two of the other contributions to our Topical Collection take steps towards doing so.

The first, Shepherd (2021), revisits the basic principles of statistical reasoning and how and when it is appropriate to use a frequentist approach in climate change science. The uncertainty of a hypothesis being tested and causal reasoning are not generally part of the logic of frequentist statistics, but are part and parcel of scientific practice in climate change science, so they need to be included in the treatment of uncertainty. Moreover, in climate change science, there is a lot of knowledge of the physical principles that govern atmospheric and ocean dynamics, but comparatively little data of the variables the evolution of which is predicted. This situation is opposite to the conditions usually present where frequentist statistics is warranted (i.e. lots of data, little prior knowledge). So, null hypothesis statistical testing (NHST) may not be the appropriate statistical foundation for probabilistic thinking for many of the cases in climate change science where these conditions materialise. Shepherd discusses three examples where NHST is applied inappropriately (the global warming “hiatus”, the Arctic amplification of global warming, and extreme event attribution), and investigates the inferential fallacies that can result as a consequence. The second part of the paper continues to discuss how the explicit consideration of the Bayes factor (but not necessarily an endorsement of a full-fledged Bayesian approach to statistics) may be a better tool to resolve some of the issues that arise in NHST as it better addresses the epistemic condition in which climate scientists find themselves when analysing model data to produce climate change knowledge.

In a similar vein, Katzav et al. (2021) comment on how knowledge - in this case specifically about future climate - is derived and represented in the physical climate change science community, and point to specific possible pitfalls of the use of full or partial probability distribution functions to represent such knowledge. The authors identify three different ways of understanding what type of knowledge probabilities represent: “objective probabilities”, which are a measurable quantity such as the probability of a coin flip having a particular outcome; “subjective probabilities”, which are degrees of belief; and “evidential probabilities”, which represent the probability of a hypothesis given the evidence available for it. While the authors argue that when these types of probabilities align it is appropriate to use PDFs (probability distribution functions), they also show that precise probabilities can misrepresent uncertainties of our knowledge of future climate in important ways, even when accompanied by caveats. This occurs specifically when PDFs are derived from model outputs, by incorrectly treating the range of model outcomes as a reliable indicator of real-world probabilities. The authors conclude by providing two alternatives: imprecise probabilities, where sets of PDFs can represent nuances in expert opinions, and formal possibilistic approaches to uncertainty representation.

The contributions of Shepherd and Katzav et al. raise important questions about how uncertainty is quantified when making statements about past and present and future climate. They show that current approaches are not satisfactory, and outline ways in which they may misrepresent uncertainty, joining an increasingly long history of concerns from different disciplinary perspectives (Parker 2010; Ambaum 2010; Stainforth and Calel 2020). They then provide distinct alternatives, from storylines to imprecise probabilities. These alternatives may have their own limitations in terms of quality and usability, or (like current approaches) may be of more use to some kinds of stakeholders than others. Further work in this space would benefit from the kind of integration that our workshop and the present Topical Collection have sought to achieve, bringing together epistemic and practice-oriented questions to explore the potential value in other representations and conceptualisations of uncertainty about climatic futures.

## Bridging the uncertainty divide: two paradigms and some ways forward

The previous section summarised the papers in this Topical Collection and provided an overview of some of the epistemic and practical issues that arise in the context of evaluating and managing the quality of forward-looking regional climate information. In this section, we take a broader view of the current state of scientific practice in the field of climate information, and make some recommendations based on the aspects of quality we have described.

Uncertainty in climate information can be a limiting factor on its use for decision support, and hence a key component of quality. But understanding varies, both of uncertainty itself and of how to deal with it scientifically. We currently see a paradigmatic difference between two conceptualisations of uncertainty, how it should inform future efforts in modelling practices, and its consequences for considerations about quality. We characterise the first paradigm as a credibility-first approach which prioritises *reduction* of uncertainty, and the second paradigm a salience-first approach which prioritises *assessment* of uncertainty.

### Credibility-first: Reduction of uncertainty as a means for achieving higher quality

The first paradigm is based around the *reduction of uncertainty*, by means of improved observations, better models, higher resolution, more effective data assimilation, larger computers and so on. This perspective is predominant in the physical and computational end of climate science. The view assumes that such a reduction in uncertainty is possible, and further, that it will necessarily improve the quality of climate information - and hence its utility for decision support - and implicitly assumes either that uncertainty is the limiting factor in information provision, or that other dimensions of quality are not the domain of science, or that they are not in scope (somebody else’s problem). The **credibility** dimension of quality, referring to the scientific justification for information, is promoted above the other dimensions.

Exemplifying this paradigm, there is an increasingly large body of work and high-level advocacy suggesting that efforts and scientific funding on the scale of hundreds of millions of dollars should be directed towards computationally-intensive high-resolution modelling, variously termed “a CERN for Climate Change”, Earth Virtualisation Engines, km-scale modelling, and so on (e.g. Palmer and Stevens 2019; Slingo et al. 2022; Hoefler et al. 2023; and the ongoing WCRP Lighthouse activity on Digital Earth). In many of these suggestions, it is assumed that higher resolution models will lead to better predictions, a reduction of uncertainty, and hence better (higher quality) information for decision-making. Based on the foregoing discussion and the papers in our Topical Collection, we identify some quality-related concerns with this paradigm:

#### Credibility

Given that this paradigm is primarily concerned with scientific credibility, the theoretical justification supporting model-derived uncertainty ranges is of critical importance. We consider the objections of Katzav et al. (2021), which are similar to those raised by Baldissera Pacchetti et al. (2021a) in their discussions of the “theory” dimension of quality, to be unanswered. If a lot of resource is to be put into increasingly complex and high-resolution models, we should have a more credible scientific justification for the benefits. Will uncertainties in fact be reduced, or will the inclusion of a larger number of less-well-understood processes result in increased ranges of uncertain outcomes? Is it the case that ever-higher resolution will result in more accurate projections, or will they simply be more detailed? Large-scale uncertainties, such as the possible role of the Marine Ice Cliff Instability in sea level rise or the possibility of passing tipping points related to ocean circulation or ecosystem changes (Lenton et al. 2008), have first-order influence on regional climates and are not necessarily resolved by more detailed models. Regardless of the grid size, there will always be sub-grid-scale processes which must be parameterised in some form (Stone and Risbey 1990), and there is no coherent theoretical justification for any modelling scale at which we will have a sufficiently good model to inform decisions (Schär et al. 2020). Shepherd’s comments on statistical practice in climate science chime with Katzav et al.’s and both papers demonstrate a need to move away from purely statistical approaches to acknowledge the not-quite-so-ideal physical world under consideration, in order to make credible statements.

#### Salience

The credibility first paradigm assumes that overall quality, including salience for users, is inversely related to uncertainty levels. This is a one-dimensional view of stakeholder needs and collapses the many dimensions and nuances identified by Wilby and Lu in their description of the varied market for different kinds of climate services, by Bojovich et al. in their description of the challenges faced by information service providers, and by Baldissera Pacchetti et al. and Jebeile in their discussions of the quality of recent UK and Swiss national climate projections. We suggest that further consideration of the salience dimensions of quality, including consultation with stakeholders, could help direct scientific efforts, whether that be to support the justification for high-resolution modelling or perhaps to suggest that some resource would be better diverted into “tailoring” to the customer rather than making the best possible suit of clothes for a shop mannequin. Lack of salience also occurs through the possibility that different stakeholders may have very different needs - not just a lower or higher level of resolution or different simulated variables, but perhaps different ways of exploring or interacting with possible futures. For example, some users want large ensemble climate simulations to gain a better representation of the statistics of extreme events (Thompson et al. 2017), but larger ensembles necessitate a trade-off with higher resolution, given finite compute resources.

#### Legitimacy/Transparency

The question of legitimacy is partially a social question, as Jebeile notes. Baldissera Pacchetti et al. also identify shortcomings in transparency related to UK climate projections. These shortcomings are more or less common to complex models because of the difficulty of explaining complex concepts relating to the model and meta-concepts about evaluation. To make the most of increasingly complex and high-resolution models, we would need a clear and accessible narrative about their function, levels of uncertainty, intended use cases and specific limitations or boundaries.

#### Diversity

Diversity in climate models at the CMIP level is provided by having multiple modelling centres contribute to the project. However, the level of independence between models is unclear. As Katzav et al. discuss in this Topical Collection, statistical treatments cannot reasonably assume that they are a meaningful sample of an underlying distribution.

The direction of travel in the uncertainty-reduction paradigm is towards fewer larger models, with diversity provided inside the framework of those models through a systematic approach to perturbing parameters, initial conditions and so on. The assumption is that *all* relevant uncertainties can be represented in this way. We believe that this assumption requires urgent justification, especially given the concerns about salience for different stakeholder communities. A paradigm of fewer larger models also raises barriers to data accessibility, as noted by the EVE proposals, and discourages efforts towards alternate representations, pushing the available climate ensembles towards a highly structured perturbed-model ensemble and stifling possible developments of alternate model structures. We also share Katzav et al.’s concerns about the statistical meaningfulness of such an ensemble. We note that if this lack of diversity prevents a more-centralised-CMIP framework from generating probability distributions about future climate, this does not make the effort useless but it does undermine some of the key justifications (in terms of economic benefits of improved information) put forward for the large investment required.

### Salience-first: Assessment of uncertainty as a means for achieving higher quality

The second paradigm is based around the *assessment of uncertainty*, by means of experimental methods, expert elicitation, multi-model intercomparison, stakeholder consultation, and so on. This perspective is predominant in the more user-connected end of weather and climate science and at the interface of physical with social science. The view assumes that decisions need to be made before substantive uncertainty reduction can be achieved, and often implicitly assumes that issues relating to uptake and communication of scientific information are the limiting factors in information provision rather than uncertainty itself. The **salience** dimension of quality, referring to the practical utility of information for decision support, is promoted above the other dimensions.

While it is less easy to characterise a paradigm that, by definition, consists of a lot of alternative approaches, we identify the following examples:

- Transdisciplinary approaches as described by Bojovic et al. in this Topical Collection (see also, for example, overviews in Rigg and Mason 2018 and MacLeod and Nagatsu 2018).
- Non-probabilistic approaches to convey information such as those discussed by Katzav et al. in this Topical Collection.
- Storyline approaches to climate information - and note that this category in itself is heterogeneous with a variety of different ideas and aims (Baulenas et al. 2023; Baldissera Pacchetti et al. 2024).
- Approaches to anticipate or imagine ‘surprise’ (Schneider et al. 1998; Parker and Risbey 2015).
- Robust decision making and similar approaches to developing strategies that are resilient to a wide range of climate outcomes rather than optimised to a set of known probabilities (Dessai et al. 2005; Weaver et al. 2013).
- Hack and crack: hacking or deconstructing climate model scenarios to try to break them or produce arbitrarily different model outcomes by tweaking models in plausible ways? (Risbey et al. 2005).

Based on the foregoing discussion and the papers in our Topical Collection, we identify some quality-related concerns with the salience-first paradigm:

#### Credibility

Bojovic et al. describe some of the trade-offs here, given that this paradigm is particularly driven by salience: it may be that there is some kind of information or product that users really want to have, but which is scientifically not (currently) possible to provide reliably (e.g., postcode level projections of extreme wind). Clearly it would not make any sense to provide a meaningless and unreliable product just because the users want it. On the other hand, in a rapidly expanding marketplace which is still poorly regulated, there are incentives for unscrupulous players to promise this kind of information (which can be generated! ) and sell it without sufficient caveats to the users. This is an affront to the credibility of the real science and potentially a risk to its legitimacy, so cannot be ignored or tolerated.

Less controversially, the question of credibility arises in connection with the use of qualitative or semi-quantitative methods such as expert elicitation in uncertainty assessment. Methodological concerns about generating probability distributions from expert opinion are described by Katzav et al. - and we note that these concerns are similar to those raised in their exploration of model-derived probabilities.

#### Salience/Diversity

Salience is the highest priority for this paradigm and this leads naturally to a diversity of approaches: every interaction between a provider and user may result in a different kind of information transfer. But this diversity is not always positive: Bojovic et al. highlight the strain that attempting to meet many different user needs can place on information providers, who are not necessarily resourced to achieve this. Some clarity could be brought here by following the proposal of Wilby and Lu to streamline these interactions into sets of similar kinds of needs (using a tailoring metaphor of “off the peg”, “outsourced” and “bespoke”) and triaging enquiries through a careful series of initial questions rather than having an unstructured interaction.

Another issue related to salience is that those who are outside the marketplace of climate services will tend to be underrepresented in climate assessments relative to those who drive the market. Those ‘outside’ include groups of people, non-human animals, ecosystems, future generations, etc. For an equitable imagining of what climate services can be, dimensions of quality will need to go beyond user needs framed only in terms of those users who currently exist and are willing to pay - although this does beg further questions about funding and values (see, e.g., Narain 2022; and Sultana 2023). A salience-first paradigm can begin to explore these ideas and interface with critical scholarship in other domains.

#### Legitimacy/Transparency

The relatively monolithic nature of the credibility-first paradigm means that transparency is achieved, or not achieved, en masse, and that legitimacy is achieved only for those users that share the values of the credibility-first paradigm. On the other hand, the heterogeneity of the uncertainty-assessment paradigm results in an opposite problem: legitimacy and transparency must be established anew for every method or every bespoke solution, creating additional evaluative work for those involved and potentially slowing the transfer and use of information. As above, the proposal of Wilby and Lu would go a long way towards establishing legitimacy and transparency as well as helping to manage diversity.

### Bridging between paradigms

Any bridge between these two paradigms will need to take quality into consideration explicitly, beginning by addressing the trade-off between salience and credibility that Jebeile’s article identified and that we have used to frame the above discussion. The gap highlighted above is in the attitude towards uncertainty. Another element of the gap is the language used around process or model evaluation, with the credibility-first paradigm focusing on quantitative performance metrics against data, and the salience-first paradigm focusing on user feedback and adequacy for purpose.

We see a helpful resolution in understanding the limits of different kinds of models for satisfying different aims. Large-scale modelling programmes may be the best way to generate information for highly-quantitative customers who also have a willingness to pay for detailed resolution, large ensembles, and multiple models, such as insurance companies and major infrastructure projects. But projections generated in this way may simply not be very useful for complex and inherently socio-political decisions about, for example, transformative change in urban climate resilience (Mehryar et al. 2022), managed retreat from a coastline (Siders et al. 2019), or dealing with risk as a smallholder farmer (van Huysen et al. 2018). The success of an approach in one sector does not guarantee its success in others; equally, failure in one sector does not show that an approach is worthless.

Thinking about the uncertainty question specifically, this framing encourages us to consider which kinds of questions will be answered more effectively by more detailed and confident projections (“what will be the maximum temperature in London’s Hyde Park in 2080?”) and which kinds of questions are more sensitive to other considerations, once the general direction is understood (“how should Londoners change their lifestyles in response to climate change?”).

Dominant uncertainties may also limit the value of resolution; for example, a highly resolved sea level model may be able to distinguish between areas that will flood at different levels, but if the Marine Ice Cliff Instability question is unresolved, that resolution is swamped (perhaps literally) by first-order uncertainties in larger variables (global sea level contributions). Where this is the case, there is no benefit achieved (yet) by reducing second-order uncertainties.

The salience-first approach could learn lessons from the international coordination and internal quality standards of the credibility-first paradigm. Climate information and climate services are rapidly expanding and are not well-regulated or coordinated, though there are efforts in this direction, such as the Copernicus Climate Change Cervice (Yang et al. 2022) and the Climateurope2 Community Support Action (Horizon Europe grant number 101056933: https://climateurope2.eu/*).* Growing awareness of climate risk and the ongoing development of legal requirements for climate-related financial risk disclosures mean that this market will continue to expand. Salience-first approaches, where they move from the scientific world into the marketplace, would benefit from inclusive but clear regulation and/or quality standards.

## Conclusion: How can a focus on quality help?

Many of the papers in this Topical Collection have put forward their own views of what high quality climate information would look like, which we have summarised and built upon here. Three of the papers (Baldissera Pacchetti et al., Jebeile et al., Bojovic et al.) use the lens of credibility, salience and legitimacy. We have identified two paradigms of information provision which respectively take a credibility-first and salience-first approach, and have considered how each might learn from the strengths of the other, making specific recommendations.

A more nuanced collective understanding of the dimensions of information quality in climate services would help users and providers of climate information work more effectively together, avoid duplication of work, help to identify gaps in provision where further research is needed, and ultimately support more confident and effective climate adaptation decisions and policymaking.

## Data Availability

Not applicable.
